# Supplementary material for: Selecting police super-recognisers
Source: PLoS One. 2023 May 17;18(5):e0283682. doi: 10.1371/journal.pone.0283682 (PMC10191310; doi:10.1371/journal.pone.0283682)
Supplement: S1 Appendix — (DOCX) [file pone.0283682.s001.docx]

# Supplementary Materials for ‘Selecting police super-recognisers’

James D. Dunn^1^, Alice Towler^1,2^, Richard I. Kemp^1^, & David White^1^*

^1^School of Psychology, UNSW Sydney, Australia

^2^ School of Psychology, University of Queensland, Australia

## Participant Screening Test Results

Super-recognisers (n = 38) were selected from a cohort of 1600 New South Wales Police Force employees that had completed 3 screening tests via the internet on their workplace computers. Screening tests were three standardised tests of face matching and memory administered online: the Glasgow Face Matching Test (GFMT; Burton et al. 2010); the Cambridge Face Memory Test long form (CFMT+; Russell et al. 2009), and the UNSW Face Test (Dunn et al. 2020).

We applied two selection criteria to scores on these tests, identifying two overlapping groups of participants that were invited for further lab-based testing. Strict SR criteria participants had scores that exceeded 1.7 standard deviations above the mean *on all* screening tests (SR Strict, *n* = 15 identified; *n* = 11 participated)*.* Weak SR criteria participants, which included the participants in the strict criteria group, had an *average score* on the screening tests that exceeded 1.7 standard deviations above the mean (SR Weak, *n* = 54 identified; *n* = 38 participated). Supplementary Table 1 shows the recruited super-recognisers scores on each screening test.

**Supplementary Table 1.** Recruited super-recognisers scores on screening tests.

| Subject | Percent correct | | | Z score | | | | Rank & selection | |
| --- | --- | --- | --- | --- | --- | --- | --- | --- | --- |
|  | *GFMT* | *CFMT+* | *UNSW* | *GFMT* | *CFMT+* | *UNSW* | *Mean* | *Cohort Rank* | *Criteria* |
| SR01 | 100 | 89 | 79 | 1.93 | 1.72 | 3.49 | 2.38 | 4 | Strict |
| SR02 | 100 | 92 | 77 | 1.93 | 1.97 | 3.06 | 2.32 | 5 | Strict |
| SR03 | 100 | 90 | 78 | 1.93 | 1.80 | 3.21 | 2.31 | 6 | Strict |
| SR04 | 100 | 96 | 73 | 1.93 | 2.31 | 2.34 | 2.20 | 9 | Strict |
| SR05 | 100 | 89 | 76 | 1.93 | 1.72 | 2.92 | 2.19 | 10 | Strict |
| SR06 | 100 | 93 | 73 | 1.93 | 2.06 | 2.49 | 2.16 | 11 | Strict |
| SR07 | 100 | 91 | 73 | 1.93 | 1.89 | 2.49 | 2.10 | 17 | Strict |
| SR08 | 100 | 95 | 71 | 1.93 | 2.23 | 2.06 | 2.07 | 19 | Strict |
| SR09 | 100 | 91 | 71 | 1.93 | 1.89 | 2.06 | 1.96 | 24 | Strict |
| SR10 | 100 | 92 | 70 | 1.93 | 1.97 | 1.91 | 1.94 | 27 | Strict |
| SR11 | 100 | 89 | 71 | 1.93 | 1.72 | 2.06 | 1.90 | 30 | Strict |
| SR12 | 100 | 85 | 83 | 1.93 | 1.37 | 4.21 | 2.50 | 2 | Weak |
| SR13 | 100 | 85 | 83 | 1.93 | 1.37 | 4.07 | 2.46 | 3 | Weak |
| SR14 | 100 | 84 | 80 | 1.93 | 1.29 | 3.64 | 2.28 | 8 | Weak |
| SR15 | 95 | 78 | 84 | 1.41 | 0.77 | 4.36 | 2.18 | 12 | Weak |
| SR16 | 95 | 89 | 78 | 1.41 | 1.72 | 3.35 | 2.16 | 13 | Weak |
| SR17 | 95 | 95 | 75 | 1.41 | 2.23 | 2.78 | 2.14 | 14 | Weak |
| SR18 | 95 | 93 | 76 | 1.41 | 2.06 | 2.92 | 2.13 | 16 | Weak |
| SR19 | 100 | 87 | 74 | 1.93 | 1.54 | 2.63 | 2.03 | 20 | Weak |
| SR20 | 100 | 86 | 74 | 1.93 | 1.46 | 2.63 | 2.01 | 21 | Weak |
| SR21 | 100 | 88 | 73 | 1.93 | 1.63 | 2.34 | 1.97 | 23 | Weak |
| SR22 | 100 | 86 | 73 | 1.93 | 1.46 | 2.49 | 1.96 | 25 | Weak |
| SR23 | 98 | 92 | 72 | 1.67 | 1.97 | 2.20 | 1.95 | 26 | Weak |
| SR24 | 95 | 75 | 82 | 1.41 | 0.43 | 3.93 | 1.92 | 31 | Weak |
| SR25 | 98 | 79 | 77 | 1.67 | 0.86 | 3.06 | 1.86 | 33 | Weak |
| SR26 | 100 | 88 | 70 | 1.93 | 1.63 | 1.91 | 1.82 | 35 | Weak |
| SR27 | 100 | 83 | 73 | 1.93 | 1.20 | 2.34 | 1.82 | 36 | Weak |
| SR28 | 98 | 80 | 75 | 1.67 | 0.95 | 2.78 | 1.80 | 38 | Weak |
| SR29 | 95 | 83 | 75 | 1.41 | 1.20 | 2.78 | 1.80 | 39 | Weak |
| SR30 | 98 | 91 | 69 | 1.67 | 1.89 | 1.77 | 1.78 | 42 | Weak |
| SR31 | 93 | 72 | 83 | 1.15 | 0.17 | 4.07 | 1.80 | 43 | Weak |
| SR32 | 98 | 81 | 74 | 1.67 | 1.03 | 2.63 | 1.78 | 44 | Weak |
| SR33 | 95 | 87 | 73 | 1.41 | 1.54 | 2.34 | 1.77 | 45 | Weak |
| SR34 | 90 | 86 | 76 | 0.90 | 1.46 | 2.92 | 1.76 | 46 | Weak |
| SR35 | 100 | 80 | 73 | 1.93 | 0.95 | 2.34 | 1.74 | 48 | Weak |
| SR36 | 95 | 89 | 71 | 1.41 | 1.72 | 2.06 | 1.73 | 49 | Weak |
| SR37 | 98 | 89 | 69 | 1.67 | 1.72 | 1.77 | 1.72 | 50 | Weak |
| SR38 | 98 | 89 | 69 | 1.67 | 1.72 | 1.77 | 1.72 | 51 | Weak |

## Gender ratios in face tests

**Supplementary Table 2.** Female:male face ratio for each test in the main paper.

| **Test** | **Female:male face ratio** |
| --- | --- |
| GFMT | 40:60 |
| UNSW Face Test | 50:50 |
| Facial recognition candidate list test | 50:50 |
| Selfie-to-passport test | 60:40 |
| EFCT Upright | 54:46 |
| EFCT Inverted | 61:39 |
| Face and body matching test | 40:60 |
| Photoboard recognition memory test | 50:50 |
| Face-in-place recognition memory test | 50:50 |

**Full ANOVA analysis**

### EFCT

In addition to the analysis presented in the main manuscript, we also compared qualitative patterns of performance on the EFCT using a 4 x 2 x 2 mixed factors ANOVA with Group as the between-subjects factor and Study Duration (2s, 30s) and Face Orientation (upright, inverted) as the within-subjects factors. The three-way interaction between all factors was significant, *F*(3,104) = 3.51, *p* = .018, η_p_^2^ = .09. To explore this interaction we split the dataset by Face Orientation factor and performed separate 4 x 2 mixed ANOVAs for upright and inverted faces (see Supplementary Figure 1).


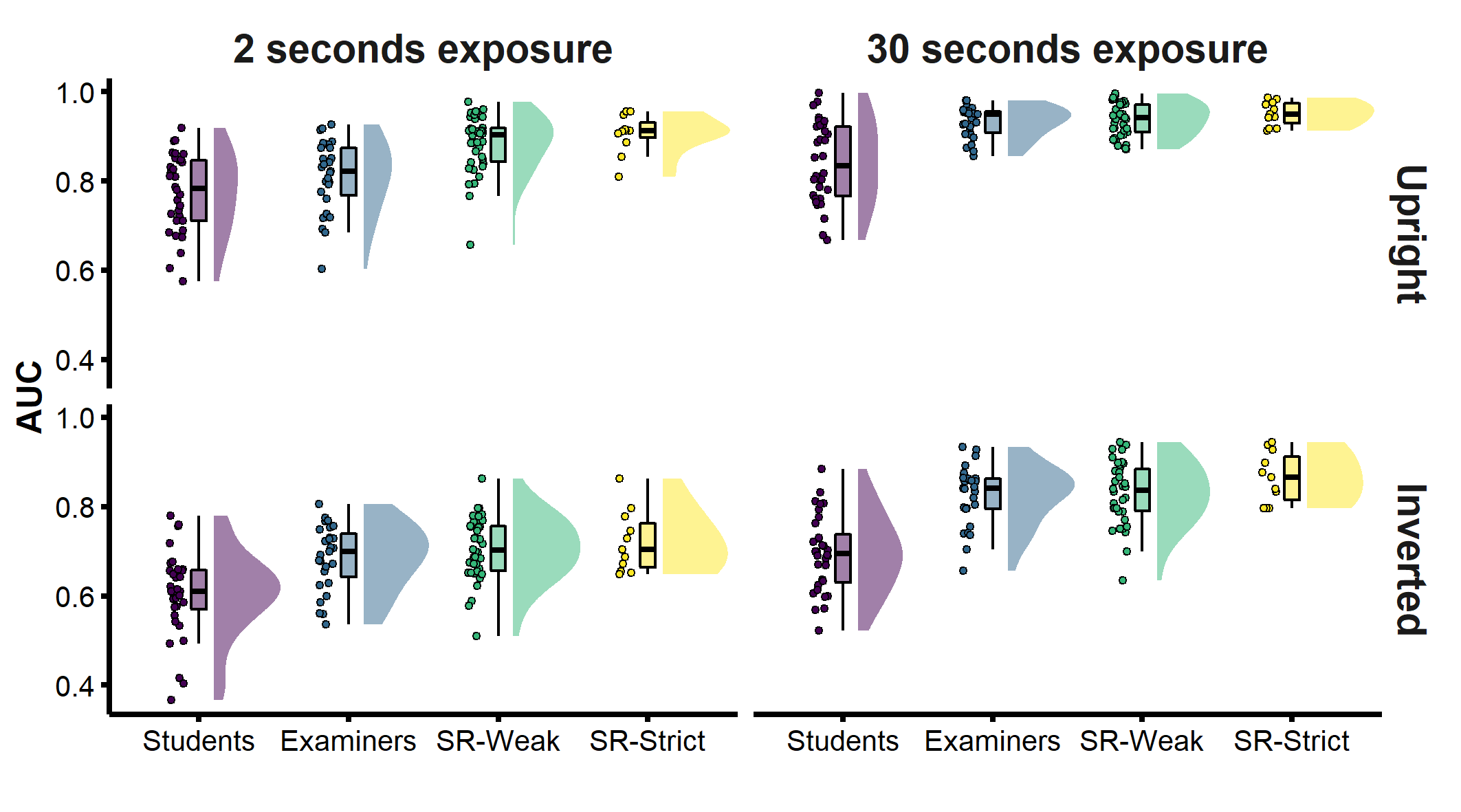


**Supplementary Figure 1**. Accuracy of participant groups on the EFCT separately for Face Orientation and Study Duration conditions.

For upright faces, there was a significant two-way interaction between Group and Study Duration (*F*(3,104) = 7.55, *p* < .001, η_p_^2^ = .18), indicating that the relative accuracy of groups varied as a function of study duration. For the 2 seconds condition, follow-up comparisons show that both SR-Weak and SR-Strict super-recogniser groups were more accurate than Examiners (SR-Weak vs. Examiners: *t*(48.3) = 3.91, *p* < .001, Cohen’s *d* = 1.02; SR-Strict vs. Examiners: *t*(32.6) = 4.71, *p* < .001, Cohen’s *d* = 1.33) and Students (SR-Weak vs. Students: *t*(56.0) = 5.86, *p* < .001, Cohen’s *d* = 1.44; SR-Strict vs. Students: *t*(35.1) = 6.51, *p* < .001, Cohen’s *d* = 1.67). For the 30 seconds condition, SR-Weak group (*M* = 0.94, *SD* = 0.04) and SR-Strict group (*M* = 0.95, *SD*  = 0.03) were both statistically equivalent to Examiners (SR-Weak vs. Examiners: *t*(57.9) = 0.67, *p* = .506, Cohen’s *d* = 0.16; SR-Strict vs. Examiners: *t*(23.9) = 1.79, *p* = .086, Cohen’s *d* = 0.58) but both were better than Students (SR-Weak vs. Students: *t*(39.6) = 5.68, *p* < .001, Cohen’s *d* = 1.45; SR-Strict vs. Students: *t*(40.7) = 6.12, *p* < .001, Cohen’s *d* = 1.38).

For inverted faces, the two-way interaction was not significant (*F*(3,104) = 1.88, *p* = .137, η_p_^2^ = .05), which accounts for the significant 3-way interaction. There was a main effect of Group (*F*(3,104) = 27.0, *p* < .001, η_p_^2^ = .44). Follow-up comparisons SR-Weak group and SR-Strict group were both statistically equivalent to Examiners (SR-Weak vs. Examiners: *t*(55.6) = 0.80, *p* = .428, Cohen’s *d* = 0.20; SR-Strict vs. Examiners: *t*(22.4) = 2.00, *p* = .057, Cohen’s *d* = 0.66) and both were better than Students (SR-Weak vs. Students: *t*(57.9) = 7.25, *p* < .001, Cohen’s *d* = 1.78; SR-Strict vs. Students: *t*(27.0) = 7.24, *p* < .001, Cohen’s *d* = 2.07). The SR-Weak group (*M* = 0.77, *SD* = 0.06) and SR-Strict (*M* = 0.79, *SD* = 0.05) group were statistically equivalent to each other (Mean difference = 0.03; *t*(19.0) = 1.46, *p* = .162, Cohen’s *d* = 0.45). There was also a main effect of Exposure duration, (*F*(1,104) = 178.1, *p* < .001, η_p_^2^ = .63) with higher accuracy in the 30 seconds condition than the 2 seconds condition.

There was also significant two-way interactions between Study Duration and Face Orientation, *F*(1,104) = 26.92, *p* < .001, η_p_^2^ = .21, between Group and Study Duration *F*(3,104) = 3.98, *p* = .010, η_p_^2^ = .10, and between Group and Face Orientation, *F*(3,104) = 2.97, *p* = .035, η_p_^2^ = .08. There were also significant main effects for Group, *F*(3,104) = 29.85, *p* < .001, η_p_^2^ = .46, Study Duration, *F*(1,104) = 240.19, *p* < .001, η_p_^2^ = .70, and Face Orientation, *F*(1,104) = 548.23, *p* < .001, η_p_^2^ = .84. As these effects were all qualified by the three-way interaction, we did not pursue follow up comparisons for any of these other effects.

### Face-in-place recognition memory test

We analysed the Hit rate on the Face-in-place recognition memory test using a 2x2 ANOVA with Group as the between-subjects factor and Context (Same Scene, Different Scene) as the within-subject factor. There was a significant main effect of Group, with a higher hit rate in super-recognisers (M = 64.3; SD = 16.5) compared to students (M = 49.6; SD = 14.7), *F*(1, 56) = 14.05, *p* < .001, η_p_^2^ = .20. The main effect for Context was marginally significant, with greater hit rate for Different Scene than Same Scene, Mean difference = 4%; *F*(1, 56) = 4.03, *p* = .049, η_p_^2^ = .07. The interaction between Context and Group was not significant, *F*(1, 56) = 1.89, *p* = .175, η_p_^2^ = .03.

Comparison of the correct rejection rate shows it is significantly higher for the SR-Weak group than students, *t*(35.7) = 2.28, *p* = .028, Cohen’s *d* = 0.66.

## Comparison of SR-Strict to norms on boundary tests

### Face and body matching test

Accuracy was analysed in a 2x(2) mixed factor ANOVA with Group (Students, SR-Strict) as the between-subjects factor and Available information (Face only, Body only) as the within-subjects factors. There was a significant main effect of group, as SR-strict group was more accurate than the Students, *F*(1,55) = 35.6, *p* < .001, η_p_^2^ = .39. Importantly, there was a significant interaction between Group and Available information, *F*(1,55) = 5.34, *p* = .025, η_p_^2^ = .09. Simple main effects show this interaction is driven by SR-strict having a larger difference in accuracy to Students in the Face only condition, Mean difference = 27.3%, *t*(35.1) = 8.11, *p* < .001, Cohen’s *d* = 1.78, than for the Body only condition, Mean difference = 16.8%, *t*(30.2) = 7.34, *p* < .001, Cohen’s *d* = 1.70.

### Photoboard recognition memory test

Performance on this task was analysed separately for Hit rate (correctly identifying learned faces) and Correct Rejection rate (correctly identifying not learned faces). Hit rate was analysed in a 2x2 mixed factor ANOVA with Group (Students, SR-Strict) as the between subjects factor and Number of study images (1, 8) as the within subjects factor. There was a significant main effect of Group, with SR-Strict having a higher hit rate than Students, *F*(1,32) = 5.40, *p* = .027, η_p_^2^ = .14. There was also a significant main effect for the number of study images, with Hit rate higher for faces studied with 8 faces compared to 1, *F*(1,32) = 33.3, *p* < .001, η_p_^2^ = .51. The interaction between factors was not significant, *F*(1,32) = 0.13, *p* = .720, η_p_^2^ = .00. Comparison of the Correct Rejection rate shows no differences between groups, *t*(23.9) = 1.53, *p* = .138, Cohen’s *d* = 0.52.

### Face-in-place recognition memory test

Performance on this task was analysed separately for Hit rate (correctly identifying learned faces) and Correct Rejection rate (correctly identifying not learned faces). Hit rate was analysed in a 2x2 mixed factor ANOVA with Group (Students, SR-Strict) as the between-subjects factor and context (Same Scene, Different Scene) as the within-subjects factor. There was a significant main effect of Group, with SR-Strict having a higher hit rate than Students, *F*(1,29) = 16.2, *p* < .001, η_p_^2^= .36. There was no significant main effect for context, *F*(1,29) = 0.56, *p* = .460, η_p_^2^ = .02. The interaction between factors was also not significant, *F*(1,29) = 3.40, *p* = .076, η_p_^2^ = .10. Comparison of the Correct Rejection rate shows SR-Strict had more correct rejections than Student controls, *t*(29.0) = 3.31, *p* = .002, Cohen’s *d* = 0.99. Finally, we also compared participants’ accuracy in identifying whether the scene was consistent between study and test for each face. Comparison of Scene Recognition accuracy shows no significant differences between Groups, *t*(18.1) = 0.14, *p* = .890, Cohen’s *d* = 0.05.

### UNSW House Test

Comparison of accuracy on the UNSW House Test shows the SR-Strict group (M = 87%, SD = 3.4) was significantly more accurate than Control participants (M = 70.2, SD = 10.0), *t*(33.4) = 7.34, *p* < .001, Cohen’s *d* = 1.94.

# References

Burton, A. M., White, D., & McNeill, A. (2010). The Glasgow Face Matching Test. *Behav Res Methods, 42*(1), 286-291. <https://doi.org/10.3758/BRM.42.1.286>

Dunn, J. D., Summersby, S., Towler, A., Davis, J. P., & White, D. (2020). UNSW Face Test: A screening tool for super-recognizers. *PLoS One*, 15(11), e0241747. <https://doi.org/10.1371/journal.pone.0241747>

Russell, R., Duchaine, B., & Nakayama, K. (2009). Super-recognizers: people with extraordinary face recognition ability. *Psychon Bull Rev*, *16*(2), 252-257. <https://doi.org/10.3758/PBR.16.2.252>
