# Supplementary material for: Selecting police super-recognisers
Source: PLoS One. 2023 May 17;18(5):e0283682. doi: 10.1371/journal.pone.0283682 (PMC10191310; doi:10.1371/journal.pone.0283682)
Supplement: Attachment — Submitted filename: SuperRecognisers_NSWPF_responseToReviewersR2.docx [file pone.0283682.s003.docx]

**James D. Dunn, Alice Towler, Richard I. Kemp, and David White**

**Author response to editor and reviewer’s manuscript PONE-D-22-32244R1**

**Selecting police super-recognisers**

**Author reply is denoted by ** and bold text.** **All page and line references refer to the tracked changes version.**

**Reviewer 1**

**Reviewer 1 did not have additional comments on this revision.**

**Reviewer 2**

Some of my previous comments have been not considered by the Authors, I would like to suggest a further effort in considering them. Specifically, from my previous points:

Previous point 1. Sex difference should be at least named in the manuscript:

- A personal reply has been added in the letter, but Point 1 is anyway omitted in the manuscript. I suggest inserting this point also in the discussion, also by inserting the explanations provided in the letter.

****We have now added an additional sentence describing these sex differences in the manuscript (Participant’s line 679) and included female:male ratios of faces in the tests to Supplementary Materials (Supplementary Table 2).**

Previous point 2. A further citation on hemispheric asymmetries for face recognition and spatial manipulation: <https://www.ncbi.nlm.nih.gov/pmc/articles/PMC4671171/>

- I understand that hemispheric asymmetries are not crucial for the present study, but I believe the suggested citation can be interesting for a potential reader of this manuscript. Obviously, the Authors are free to decide whether this reference is of interest – my personal opinion is favourable to this option.

****We continue to believe that this paper is not sufficiently relevant to our study to warrant inclusion in the reference list because it is not about individual differences in face recognition ability.**

Previous point 3. To facilitate the reading of the manuscript, the set of paradigms could be numbered:

- I still believe that the numbering would make it much easier to follow the manuscript.

****We have now numbered the subsections of Method & Results and refer to these in the final paragraph of the introduction.**
